# Septic encephalopathy in the elderly – biomarkers of potential clinical utility

**DOI:** 10.3389/fncel.2023.1238149

**Published:** 2023-09-07

**Authors:** Sandra Schütze, Douglas A. Drevets, Simone C. Tauber, Roland Nau

**Affiliations:** ^1^Department of Neuropathology, University Medicine Göttingen, Georg-August University Göttingen, Göttingen, Germany; ^2^Department of Geriatrics, AGAPLESION Markus Krankenhaus, Frankfurt, Germany; ^3^Infectious Diseases, Department of Internal Medicine, University of Oklahoma HSC, Oklahoma City, OK, United States; ^4^Department of Neurology, University Medicine Aachen, Rheinisch-Westfälische Technische Hochschule Aachen, Aachen, Germany; ^5^Department of Geriatrics Evangelisches Krankenhaus Göttingen-Weende, Göttingen, Germany

**Keywords:** sepsis, plasma, cerebrospinal fluid, magnetic resonance tomography, positron emission tomography, electroencephalography, neurofilament light chains, S100B protein

## Abstract

Next to acute sickness behavior, septic encephalopathy is the most frequent involvement of the brain during infection. It is characterized by a cross-talk of pro-inflammatory cells across the blood–brain barrier, by microglial activation and leukocyte migration, but not by the entry of infecting organisms into the brain tissue. Septic encephalopathy is very frequent in older persons because of their limited cognitive reserve. The predominant clinical manifestation is delirium, whereas focal neurological signs and symptoms are absent. Electroencephalography is a very sensitive method to detect functional abnormalities, but these abnormalities are not specific for septic encephalopathy and of limited prognostic value. Routine cerebral imaging by computer tomography usually fails to visualize the subtle abnormalities produced by septic involvement of the brain. Magnetic resonance imaging is by far more sensitive to detect vasogenic edema, diffuse axonal injury or small ischemic lesions. Routine laboratory parameters most suitable to monitor sepsis, but not specific for septic encephalopathy, are C-reactive protein and procalcitonin. The additional measurement of interleukin (IL)-6, IL-8, IL-10 and tumor necrosis factor-α increases the accuracy to predict delirium and an unfavorable outcome. The most promising laboratory parameters to quantify neuronal and axonal injury caused by septic encephalopathy are neurofilament light chains (NfL) and S100B protein. Neuron-specific enolase (NSE) plasma concentrations are strongly influenced by hemolysis. We propose to determine NSE only in non-hemolytic plasma or serum samples for the estimation of outcome in septic encephalopathy.

## 1. Introduction

**Figure 1:**
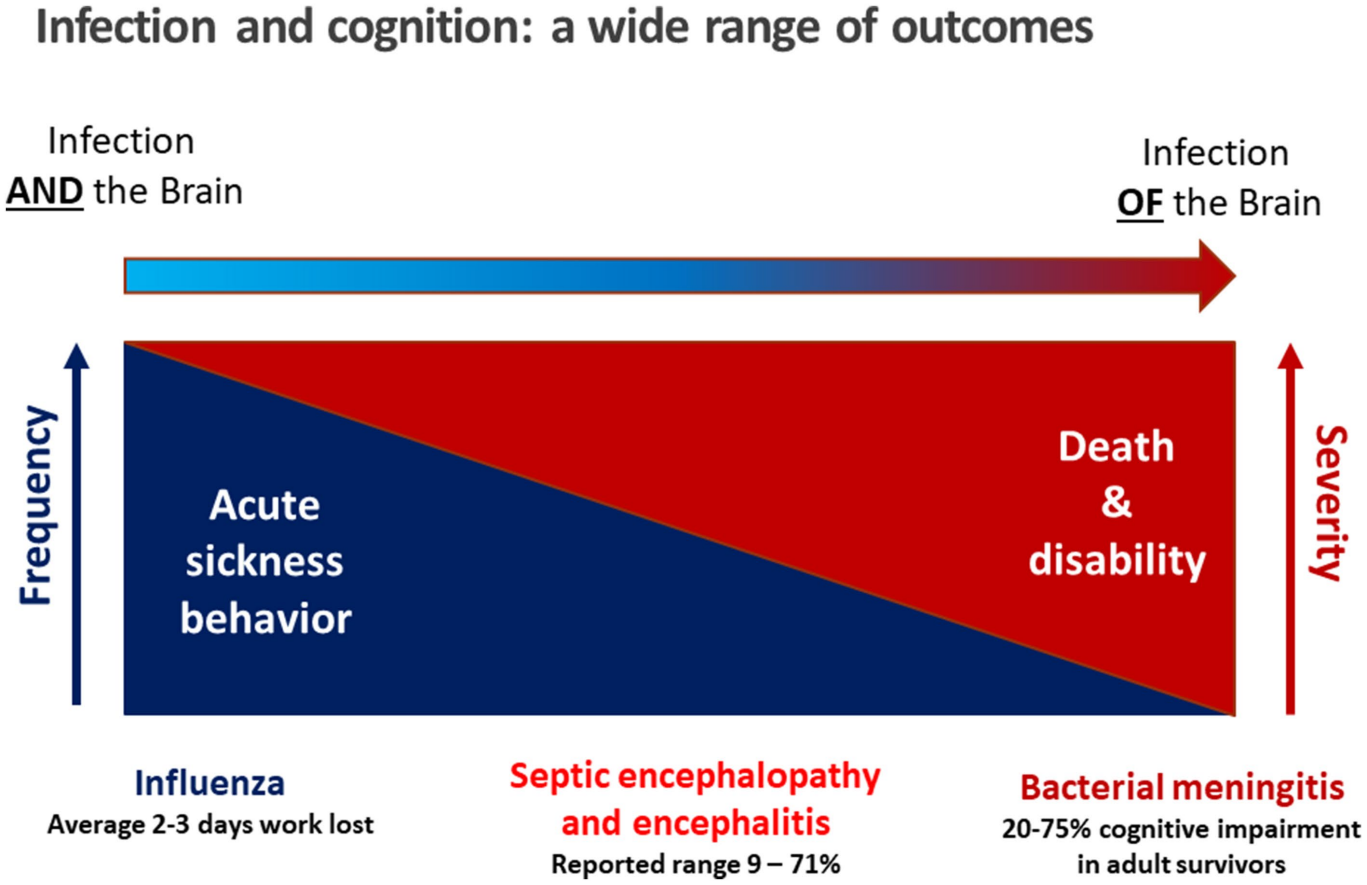
Conceptual relation between infection and cognition. During an acute, mild viral infection such as influenza, acute sickness behavior manifests as malaise, mild hypoxia, headache, and lost productivity (Zumofen et al., 2023) as a consequence of acute sickness behavior caused by a short systemic inflammatory reaction. Antiviral therapy usually is not indicated. On the other end of the spectrum when there is microbial invasion of the brain, e.g. in bacterial meningitis, pathogens and bacterial products are present in the cerebrospinal fluid and brain tissue causing direct and immune-mediated neuronal damage (Nau and Brück, 2002; Too et al., 2021). In septic encephalopathy, bacteria do not enter the brain, and neuronal injury predominantly is not caused directly by bacterial products, but by cross-talk of immune cells across the blood–brain barrier leading to microglial activation and entry of immune cells into the brain. In septic encephalitis, pathogens in low quantities enter the brain, either causing microabscesses (septic-metastatic form) or vasculitis and cerebral ischemia by infected emboli (septic-embolic form).

The brain can be affected by many different types of infections with differing degrees of severity. The mildest situation is acute sickness behavior induced by a short inflammatory burst in the systemic circulation communicated to the brain via (a) leaky regions of the blood–brain barrier in the circumventricular organs, (b) afferent fibers of the vagus nerve, and (c) direct communication across the blood–brain barrier leading to a short-term reduction of cognitive function without long-term sequelae (Perry et al., 2003; Tauber et al., 2021; Too et al., 2021; Zumofen et al., 2023). Here, the brain does not come into direct contact with the pathogen causing the acute infection or compounds produced by this pathogen (Figure 1). In contrast, the most severe end of the spectrum of involvement of the brain in infection is when the pathogen has entered the cerebrospinal fluid (CSF) or/and the brain. This is the case in bacterial or fungal meningitis, viral encephalitis and different forms of intracranial abscesses. In addition to pro-inflammatory mediators, microbes and components released by them, in particular hemolysins and free radicals, can target neurons, and their products can directly stimulate immune cells in the brain or are toxic to different cell types present in nervous tissue (Nau and Brück, 2002; Too et al., 2021).

**Figure 2:**
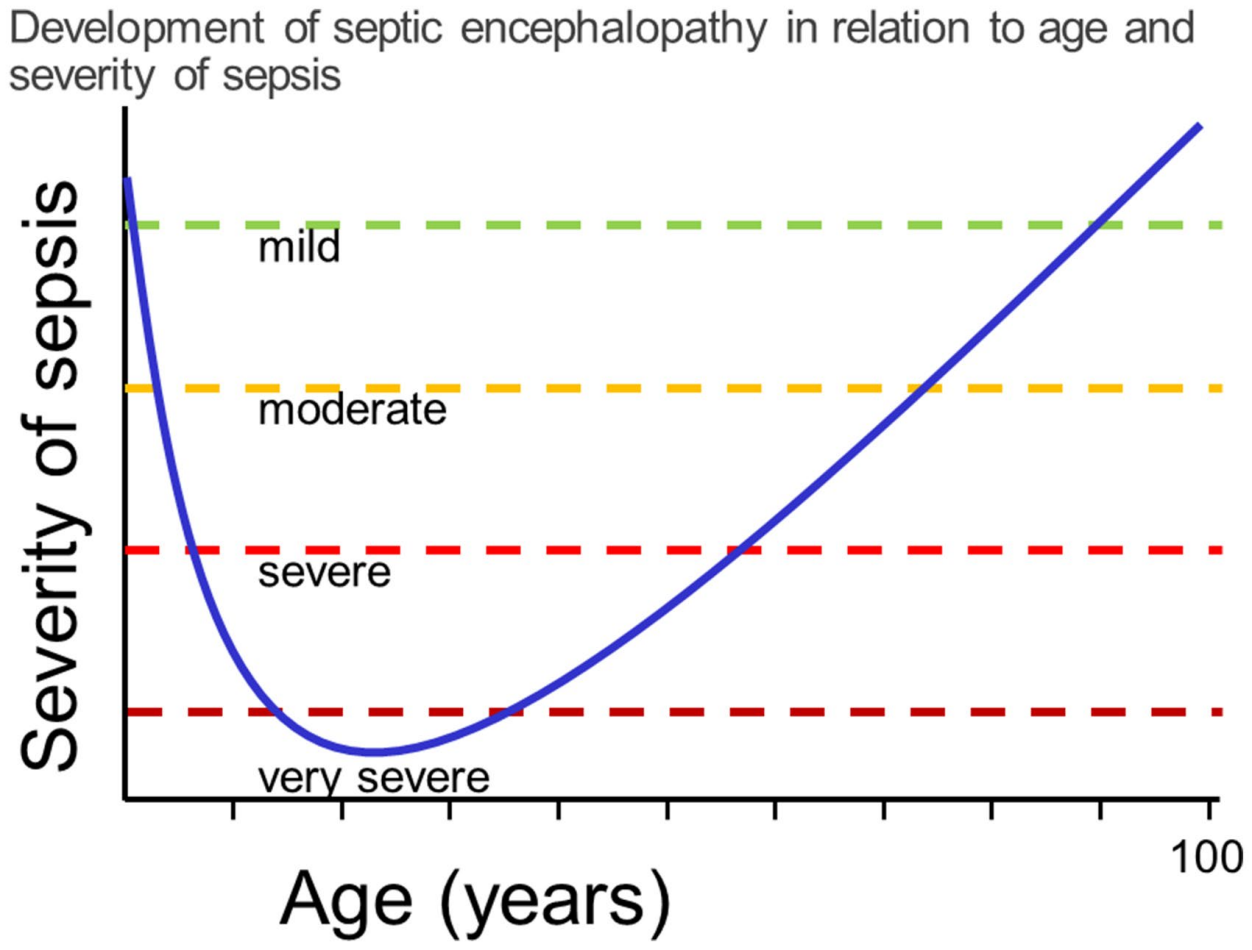
The vulnerability to septic encephalopathy depends on the cognitive reserve of the patient, which is determined by age and concomitant diseases. In the first years of life and beyond the age of 60, mild or moderate septicemia can cause septic encephalopathy. Conversely, in previously healthy adolescents and young adults, only very severe systemic infections can cause septic encephalopathy, i.e., the risk to develop septic encephalopathy is low.

Septic encephalopathy is a disease entity between these two extremes: circulating immune cells and mediators of inflammation communicate with the brain via the circumventricular organs, afferent fibers of the vagus nerve and the endothelium of the vessels in brain parenchyma, meninges and choroid plexus. The entry of bacterial products through leaks of the blood–brain barrier plays a minor role, and the pathogen itself does not enter the brain parenchyma or the CSF (Perry et al., 2003; Figure 1). Whereas the incidence of community-acquired meningitis has decreased substantially in industrialized countries over the past 50 years, the incidence of septic encephalopathy has risen due to increased numbers of susceptible persons, particularly those over the age of 50 years. The susceptibility of the brain to septic encephalopathy (or sepsis-associated encephalopathy) is age-dependent. It is high in newborns, infants and toddlers, then decreases in older children and is very low in young adults. Beyond the age of 50 years, it gradually increases due to aging of the immune system, concomitantly administered medications and co-morbidities, and a physiological age-related decline in the functional capacity of most organs (Werner and Kuntsche, 2000; Lu et al., 2022). For these reasons the incidence of sepsis in persons beyond 70 years is approximately 100 times higher than in young adults (Angus et al., 2001). Septic encephalopathy that manifests as delirium, stupor or coma has become a common problem in geriatric medicine. Whether it develops in an older person depends on the severity of the infectious challenge and the cognitive reserve, or cognitive resilience, of the individual (Cizginer et al., 2017; Figure 2). In the pathophysiology of septic encephalopathy in advanced age, the stimulation of microglial cells that are pre-activated by amyloid-β, α-synuclein and other endogenous compounds deposited in the brain during neurodegenerative diseases, and to an extent also during healthy aging, also play a key role (Perry et al., 2003).

**Figure 3:**
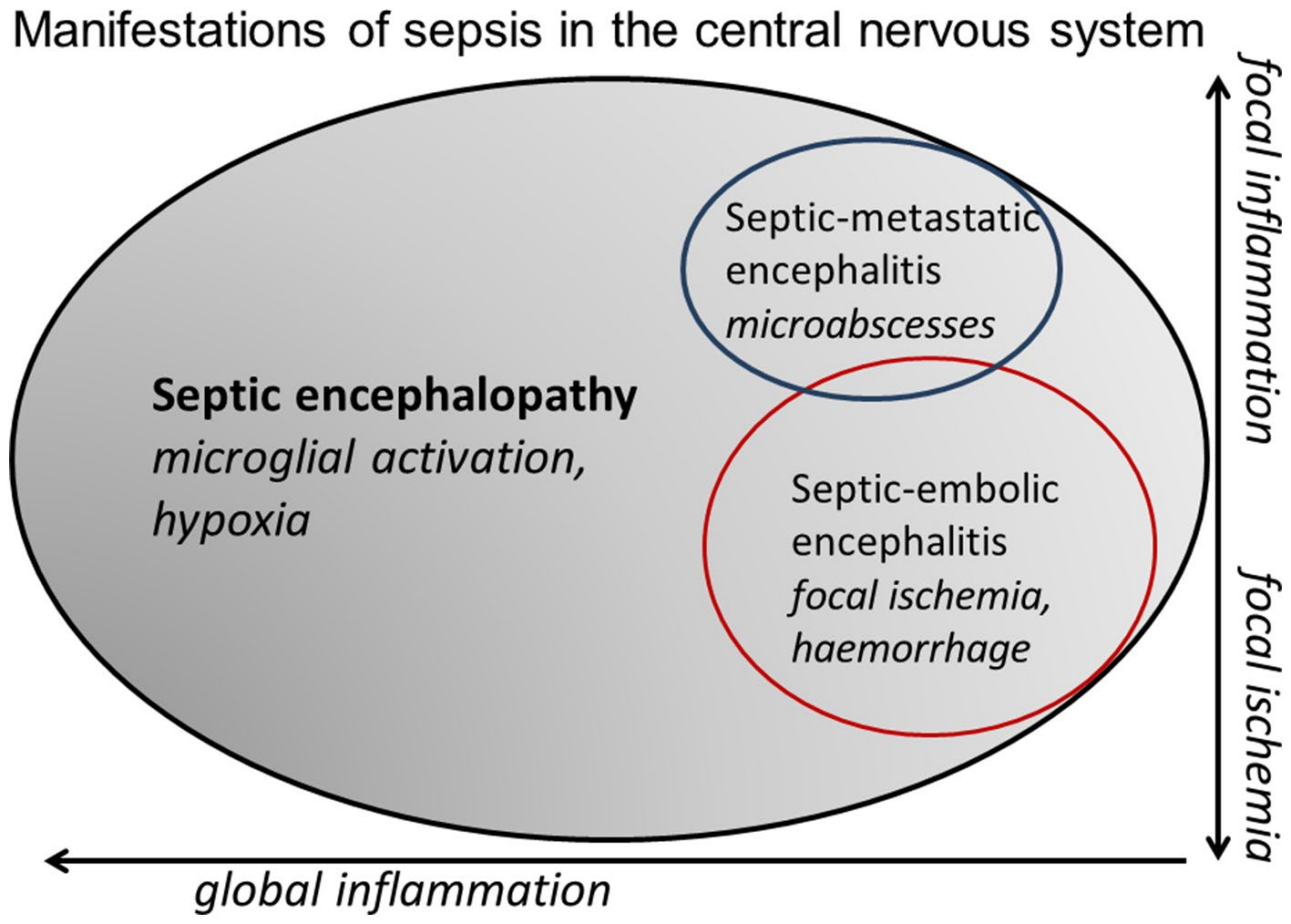
Venn diagram illustrating the continuum between septic encephalopathy (microglial activation and tissue hypoxia as a consequence of septic shock predominate), septic-embolic (ischemic lesions caused by septic emboli predominate) and septic-metastatic encephalitis (microabscesses predominate).

Septic encephalopathy is the prototype of an organic psychosis. In contrast to septic embolic or metastatic encephalitis, where focal neurological deficits are present and often determine the clinical picture (Tauber et al., 2017, 2021; Figure 3), the main clinical manifestation of moderate septic encephalopathy is delirium, and of severe encephalopathy stupor or coma. In a large retrospective study comprising 2,513 patients with sepsis at ICU admission, potentially modifiable factors contributing to septic encephalopathy were acute renal failure, hypo- and hyperglycemia, hypercapnia and hypernatremia (Sonneville et al., 2017). One of the most delightful outcomes for a clinical geriatrician is the rapid recovery of some patients after appropriate antimicrobial treatment and fluid and electrolyte management. Unfortunately, after septic encephalopathy many persons do not reach their pre-infection cognitive level. Animal studies suggest a time-dependent recovery from anxiety and depression (Dal-Pizzol et al., 2021), whereas cognitive impairment apparently persists (Savi et al., 2021). Patients surviving septic encephalopathy have a higher risk of developing a neurodegenerative disease than persons without sepsis or, when a neurodegenerative disease already is present, experience an acute exacerbation or acceleration of their disease (Perry et al., 2003). Similarly, recent studies indicate that adults who survive a variety of infections severe enough to require hospitalization have a significantly increased risk of developing dementia over the next 10–20 years (Sipilä et al., 2021; Bohn et al., 2023). Severe sepsis in the elderly approximately triples the risk of the development of moderate to strong new cognitive impairment in this population (Peters van Ton et al., 2022; Piva et al., 2023). These findings in humans are recapitulated in a mouse model of intraperitoneal infection with *Listeria monocytogenes* followed by amoxicillin treatment (Cassidy et al., 2023). Here, systemic infections by neuroinvasive wild-type bacteria, as well as by non-neuroinvasive ∆*hly* mutants induced cognitive impairment manifest 4 months after infection. Similar to findings in patients (Sipilä et al., 2021), cognitive deficits were more profound after infection with neuroinvasive *L. monocytogenes,* and correlated significantly with the retention of CD8+ T-lymphocytes in the brain. The presence of cognitive decline after non-neuroinvasive infection, which did not lead to retained white blood cells in the brain, suggests that neuroinvasive and non-neuroinvasive infections may trigger cognitive decline via different mechanisms (Cassidy et al., 2023). In addition to CD8+ T-cell instigated neuroinflammation, another possible source of injury is through alterations in endothelial function. For example, the moment-to-moment adjustment of cerebral blood flow to match the increased demands of active brain regions, known as neurovascular coupling, was impaired in a geriatric *L. monocytogenes* sepsis model prior to the development of cognitive impairment (Csipo et al., 2021).

Delirium is defined as “a disturbance in attention (i.e., reduced ability to direct, focus, sustain, and shift attention) accompanied by reduced awareness of the environment” (American Society for Psychiatry, 2022). It has a range of severities and typically presents with fluctuating symptoms. Therefore, repeated clinical assessments are necessary to quantify its severity. Two widely applied rapid methods are the general ward confusion assessment method (CAM) and the confusion assessment method in the intensive care unit (CAM-ICU). Usually measurements are performed 3 times daily in order to assess the mental status of patients at risk for, or already suffering from delirium. CAM (applied on general wards) and CAM-ICU (applied on intensive care wards) in several studies showed a high sensitivity and specificity (CAM: 94–100%, 89–95%; CAM-ICU: 97–100%, 89–100%) (data summarized by Yuechen et al., 2023). The severity of delirium also can be estimated by scales, e.g. the CAM-S (Khan et al., 2017).

Unlike septic-embolic or -metastatic encephalitis, which can be visualized by cranial computer tomography (CCT) or – more sensitive and specific, but less suitable in agitated geriatric patients - cranial magnetic resonance imaging (cMRI) without and with contrast enhancement (Tauber et al., 2017, 2021), septic encephalopathy often cannot be detected or visualized by routine imaging with CCT or cMRI. Therefore, the clinician is confronted with the problem to substantiate the diagnosis “septic encephalopathy” in order not to overlook other diagnoses requiring more specific treatment.

The pathophysiology of septic encephalopathy is complex involving cross-talk across the blood–brain barrier, microglial activation, endothelial swelling, a disruption of the blood–brain barrier, vasogenic edema, leukocyte migration, impairment of cerebral autoregulation and perfusion, excitotoxicity, mitochondrial dysfunction and neuronal and axonal injury. The most extreme manifestation of septic encephalopathy occurs in deep septic shock with bilateral hippocampal necrosis as a consequence of watershed infarctions (Tauber et al., 2017). Pathophysiological mechanisms have been discussed in detail in several recent publications (Perry et al., 2003; Tauber et al., 2017, 2021; Robba et al., 2018; Too et al., 2021; Sekino et al., 2022; Dumbuya et al., 2023; Barichello et al., 2023; Piva et al., 2023). This review will focus on biomarkers of septic encephalopathy which have already been successfully applied in clinical practice and which show promise as prognostic markers in older patients.

## 2. Indicators of sepsis and septic encephalopathy

Sepsis affects multiple organ systems, and many biomarkers are produced/released by cells not belonging to the central nervous system (CNS). Therefore, general biomarkers of sepsis alone or in combination generally do not display sufficiently high levels of specificity to discriminate between sepsis without and with septic encephalopathy. For this reason, it is crucial to differentiate between biomarkers of sepsis from those of septic encephalopathy. Specific markers of septic encephalopathy are compounds produced/released exclusively by the brain. For sepsis, a large variety of biomarkers has been identified, including soluble pattern recognition molecules (PRMs), elements of the complement system, several cyto- and chemokines, damage-associated molecular patterns (DAMPs), non-coding RNAs, microRNAs, cell membrane receptors, cell proteins, metabolites, and soluble receptors (Barichello et al., 2022).

### 2.1. Biomarkers and symptoms of sepsis

#### 2.1.1. Fever

Fever is not a biomarker, but a symptom of infection including sepsis already known to Galenos of Pergamon. Because of immune-aging, fever is not present in all aged patients with sepsis. In a large retrospective study among 15,574 sepsis patients (mean age 70.3 years), upon admission 9.7% of the patients were hypothermic, 73.6% normothermic, and 16.8% hyperthermic (Baek et al., 2022). Conversely, fever in older patients can be present due to localized bacterial infections without progression to sepsis as well as in a variety of viral infections. In sepsis, the 90-day mortality rate was inversely correlated with the body temperature. The negative correlation between body temperature and mortality was also observed in patients aged < 75 years, but was even stronger in patients ≥ 75-years of age (Baek et al., 2022).

#### 2.1.2. Indicators of sepsis and systemic inflammation in blood established in clinical routine

In order to rule out other concomitant diseases and assess the involvement of other organs in septic encephalopathy, the following routine parameters must be performed upon hospital admission (and often repeatedly during the course of the disease):

##### 2.1.2.1. Complete cell count with differential

Usually blood leukocytes are elevated during bacterial and fungal septicemia. In patients with suspected infection requiring intensive care, leukopenia was associated with an increased mortality compared to leukocytosis (Belok et al., 2021).

##### 2.1.2.2. Blood glucose

Hypoglycemia can mimic all symptoms of septic encephalopathy, hyperglycemia can cause stupor and coma (Umpierrez and Korytkowski, 2016).

##### 2.1.2.3. Arterial blood gas analysis including measurements of oxygen and carbon dioxide pressure, pH, lactate and base excess

Elevated lactate concentrations in plasma are correlated to in-hospital mortality. Patients with lactate concentrations >4 mmol/L, in the absence or presence of hypotension, have a higher in-hospital mortality than patients with intermediate (2–3 and 3–4 mmol/L) or normal lactate concentrations (Casserly et al., 2015). Metabolic acidosis can induce confusion, malaise and lethargy and thereby resemble septic encephalopathy (Cruz-Flores, 2021; Mahmood et al., 2023).

##### 2.1.2.4. Electrolytes

Hypo- and hypernatremia and hypercalcemia can mimic and aggravate septic encephalopathy (Riggs, 2002; Cruz-Flores, 2021).

##### 2.1.2.5. Assessment of renal and liver function by creatinine, urea, liver enzymes, and of the coagulation status (prothrombin time, partial thromboplastin time, fibrinogen)

Uremic and hepatic encephalopathy can mimic septic encephalopathy, and abnormalities of coagulation can increase the risk of intracranial hemorrhage or focal or generalized hypoperfusion (Iacobone et al., 2009; Yuechen et al., 2023).

##### 2.1.2.6. C-reactive protein (CRP)

**Figure 4:**
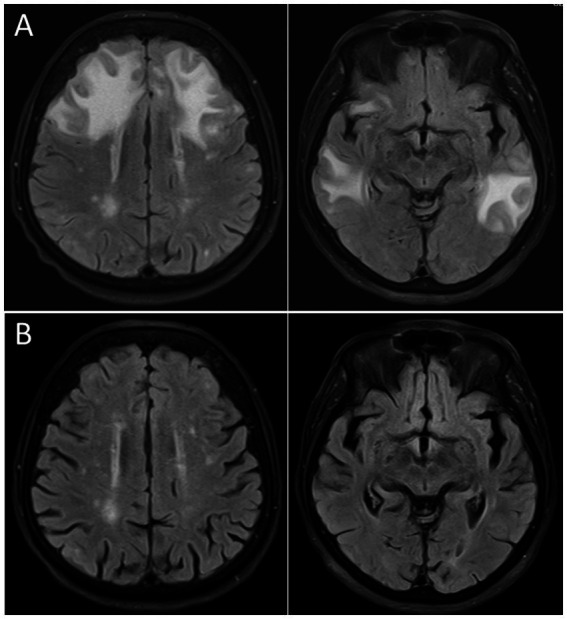
Cranial magnetic resonance imaging (cMRI), fluid attenuated inversion recovery (FLAIR) sequence, of an 87 years old woman presenting with severe delirium upon *Escherichia coli* urosepsis. **(A)** Distinct vasogenic edema in the frontal and temporal lobe compatible with a posterior reversible encephalopathy syndrome (PRES) 2 days after onset of clinical symptoms. **(B)** Follow-up imaging 5 weeks later demonstrating complete resolution of the vasogenic edema.

CRP is an acute phase protein released by the liver. The rise of the CRP concentration in sepsis is primarily induced by interleukin (IL)-6 and IL-1β (Barichello et al., 2022). CRP can activate the complement system, recruit leukocytes to the site of inflammation, and mark pathogens for phagocytosis by identifying and binding foreign molecules on the cell walls of pathogens. Combining CRP with the quick sequential organ failure assessment (qSOFA) probably improves the accuracy of qSOFA alone in identifying patients at risk of dying in hospital from sepsis (Zacharakis et al., 2023). In a retrospective study on the posterior reversible encephalopathy syndrome (PRES, one of the radiological manifestations of septic encephalopathy) in 151 patients, both in univariate and multivariate analyses, higher levels of CRP were associated with in-hospital death (Siebert et al., 2017) (Figure 4). High CRP plasma concentrations can predict prolonged periods of acute brain dysfunction (McGrane et al., 2011).

##### 2.1.2.7. Procalcitonin (PCT)

PCT is the precursor peptide of calcitonin, which regulates calcium homoeostasis. PCT is released ubiquitously by parenchymal cells (mainly in the liver, the kidney, adipose tissue and muscle) in response to microbial toxins and to several pro-inflammatory cytokines, in particular IL-1β, tumor-necrosis factor (TNF)-α and IL-6 (Falcone et al., 2023). PCT can discriminate bacterial and fungal infections with access to the systemic circulation from viral infections. PCT, however, is less suitable to assess the severity of sepsis, because the values measured strongly depend on the pathogen: they are highest in Gram-negative, intermediate in Gram-positive bacterial, and lowest in fungal septic infections (Leli et al., 2015).

##### 2.1.2.8. Cytokines and chemokines

In early septic shock, in addition to well established biomarkers of infection (C-reactive protein, ferritin, and procalcitonin levels), the cytokines IL-1β, IL-2R, IL-6, IL-8, IL-10, and TNF-α in plasma strongly increase. Compared to survivors, non-survivors had higher plasma concentrations of cytokines (IL-6, IL-8, and IL-10), but lower levels of IgM and the complement fractions C3 and C4, lymphocyte and CD4+, and CD8+ T cell counts. Low IgM or C3 plasma concentrations and low lymphocyte or CD4+ T cell counts in blood were independent risk factors for mortality (Tang et al., 2023).

Some inflammatory markers appeared to be useful to differentiate between septic encephalopathy and other causes of delirium: higher levels of vascular cellular adhesion molecule-1, intercellular adhesion molecule-1, platelet-derived growth factor-AB/BB and RANTES [“regulated on activation, normal T cell expressed and secreted”, chemokine C-C motif ligand 5 (CCL5)] and of brain-derived neurotrophic factor were observed in septic encephalopathy patients compared to patients with delirium by other causes (Tomasi et al., 2017). IL-8 levels in the blood were associated with delirium in patients with infection, whereas IL-10 and amyloid-β 1–42 and 1–40 blood concentrations were elevated in delirium in non-infected patients (van den Boogaard et al., 2011). In septic encephalopathy, coma patients (*n* = 18) displayed higher IL-6, TNF-α and IL-12 plasma concentrations than delirium patients (*n* = 64) (Orhun et al., 2019). Since circulating cytokines influence the endothelium and the parenchymal cells of many organs, their concentrations are rather general indicators of the severity of sepsis and no specific biomarkers of septic encephalopathy. The cytokines IL-6, IL-8, IL-10 and TNF-α are established for the assessment of outcome in sepsis (Ishikawa et al., 2021; Xiao et al., 2022). When septic encephalopathy was compared with sepsis without encephalopathy, plasma concentrations of brain-derived neurotrophic factor (BDNF) were higher in septic encephalopathy patients, whereas RANTES and IL-10 concentrations were lower in patients with than in patients without encephalopathy suggesting BDNF as a possible biomarker of septic encephalopathy (Tomasi et al., 2017).

##### 2.1.2.9. Micro-ribonucleic acids (MicroRNA)

MicroRNAs are non-coding RNAs with a typical length of 21–23 nucleotides. They induce messenger RNA (mRNA) degradation or repress protein translation by binding to target mRNAs thereby playing a role in gene silencing and are up- and down-regulated according to the progression of septic encephalopathy. They are relatively stable, present in many body fluids and probably able to pass the impaired blood–brain barrier. Therefore, they have been proposed as biomarkers for septic encephalopathy. High plasma concentrations of microRNA-370-3p were observed in patients with septic encephalopathy, but not in patients with sepsis or uremia alone (Visitchanakun et al., 2020). MicroRNA-29a, used for the surveillance of patients with pancreatic tumors, was elevated in patients with septic encephalopathy compared to patients with other forms of encephalopathy, and high concentrations were associated with a poor prognosis (Guo et al., 2021). Previously published data on the regulation of different microRNAs in humans have been summarized recently (Antonakos et al., 2022; Yuechen et al., 2023). MicroRNA-133a and microRNA-25 at present appear most promising as markers of the severity of sepsis (Ehler et al., 2022). Brain-specific microRNA, such as microRNA-124, microRNA-128, microRNA-137, microRNA-219, microRNA-335 and microRNA-338 (Suster and Feng, 2021; Li et al., 2023), to our knowledge have not been studied in sepsis yet.

##### 2.1.2.10. Natriuretic peptides

C-type natriuretic peptide (CNP) is released by endothelial cells as a protective mechanism in response to cardiovascular disease. It is expressed in the vascular endothelium of many organs including the brain (Koch et al., 2011) and released during encephalopathy (Ehler et al., 2019b). The amino-terminal propeptide of the C-type natriuretic peptide (NT-proCNP) was compared as a biomarker of septic encephalopathy with neuron-specific enolase (NSE) and S100B protein in 12 patients diagnosed septic encephalopathy and 9 non-septic control patients without encephalopathy. NT-proCNP concentrations in plasma were higher in septic encephalopathy compared to control patients, whereas plasma NSE levels were not significantly different between both groups. Although not specific for brain endothelium, NT-proCNP plasma levels might, unlike CSF concentrations, indicate neurological impairment in septic encephalopathy (Ehler et al., 2019b).

In a small prospective study, N-terminal probrain natriuretic peptide (NT-proBNP) values at 72 h were an independent predictor of mortality in patients with sepsis (Biswas et al., 2022). NT-proBNP has not been studied in septic encephalopathy.

### 2.2. Specific indicators of neuronal injury in sepsis

Biomarkers derived exclusively from cells of the CNS are the only indicators able to differentiate neural injury from damage to other organs. Products of neural cells can reach the bloodstream across the blood-brain barrier, which in septic encephalopathy often is more permeable to larger molecules than in health, or by CSF bulk flow. In order to quantify neural injury, concentrations of these compounds in blood are influenced by the severity of damage as well as by disease-related variations of the permeability of the blood–brain barrier and CSF flow (Djukic et al., 2022).

#### 2.2.1. Neuron-specific enolase

Neuron-specific enolase (NSE) is a cell-specific isoenzyme of the glycolytic enzyme enolase. Vertebrates possess 3 isozymes of enolase, expressed by different genes: enolase-α is ubiquitous; enolase-β is muscle-specific and enolase-γ (NSE) is neuron-specific (Isgrò et al., 2015). Since NSE is also present in erythrocytes and thrombocytes, either hemolytic samples must be excluded or a NSE correction method has to be employed for hemolyzed samples (Lauwers et al., 2022). Since practices to report the hemolysis index are poorly standardized across different manufacturers of assays and laboratory equipment (Simundic et al., 2020), we suggest to use only samples with no detectable hemolysis for NSE determination in plasma or serum. In a retrospective analysis of 124 septic adults, each doubling of the NSE concentration measured in plasma upon admission was associated with a 7% increased risk of 30-day mortality and a 5% increased risk of delirium (Anderson et al., 2016). In a small study in adults, plasma NSE concentrations from twelve patients with septic encephalopathy and nine non-septic controls without encephalopathy were not significantly different (Ehler et al., 2019b). In neonates, NSE in umbilical blood was elevated in septic encephalopathy and was related to an impairment of neural development 6 months later (El Shimy et al., 2018).

#### 2.2.2. S100B

S100B is a calcium-binding protein primarily expressed by astrocytes. It can, however, be also expressed by oligodendrocytes, glial progenitor cells, cytotoxic CD8-positive T cells and natural killer cells, neutrophils and macrophages (Steiner et al., 2007, 2011; Miki et al., 2013; Rossi et al., 2023). In a meta-analysis of 28 studies with 1,401 serum samples from patients with septic encephalopathy and 1,591 serum samples from septic patients without encephalopathy, patients with septic encephalopathy had higher serum S100B concentrations than septic control patients. Septic patients with an unfavorable outcome displayed higher serum S100B levels than those with a favorable outcome making S100B a promising indicator of neural injury in septic encephalopathy (Hu et al., 2023). S100B appears to be more sensitive and specific to predict septic encephalopathy than NSE (Ehler et al., 2019a,b) possible because of the influence of hemolysis on plasma NSE concentrations. In a prospective observational study (112 patients enrolled, 48 diagnosed with septic encephalopathy), S100B (cut-off 0.131 μg/L) diagnosed septic encephalopathy with 67.2% specificity and 85.4% sensitivity (area under the receiver operating characteristic [ROC] curve 0.824), whereas NSE (cut-off 24.15 ng/mL) diagnosed septic encephalopathy with 82.8% specificity and 54.2% sensitivity (area under the ROC curve 0.664). The area under the ROC curve for the prediction of hospital mortality of S100B also was larger than that of NSE (0.730 vs. 0.590) (Yao et al., 2014).

#### 2.2.3. Glial fibrillary acidic protein

Glial fibrillary acidic protein (GFAP) is expressed by astrocytes and ependymal cells, but also in extracerebral tissues such as in glomeruli and fibroblasts of the kidney. Serum GFAP concentrations were higher in patients with septic encephalopathy than in septic patients without encephalopathy. High GFAP concentrations were an indicator of poor outcome (Wu et al., 2019). In a study on 152 patients, the serum concentrations of GFAP in patients with septic encephalopathy were higher than those in patients without encephalopathy. The area under the ROC curve of GFAP was higher than the area under the ROC curve of NSE and S100B. In sepsis, serum GFAP concentrations correlated with Acute Physiology and Chronic Health Evaluation II (APACHE II) score and inversely correlated with the Glasgow Coma Scale score and the survival rate at day 28 and at day 180 (Yan et al., 2019).

#### 2.2.4. Amyloid-β and Tau proteins

Alzheimer’s disease (AD) is characterized by an abnormal aggregation and deposition of amyloid-β peptides of different lengths into extracellular plaques and of hyperphosphorylated Tau protein (p-Tau) into intracellular neurofibrillary tangles, followed by progressive cognitive and functional decline (Andrews et al., 2023). Amyloid-β 1–40 and amyloid-β 1–42 serum concentrations did not differ significantly in patients with septic encephalopathy and healthy age-adjusted control patients (Orhun et al., 2019). Conversely, Tau protein concentrations in serum were elevated in 27 patients with septic encephalopathy compared to 82 septic patients without encephalopathy. Serum Tau and the sequential organ failure assessment (SOFA) score were independently associated with septic encephalopathy. The combined use of serum Tau protein concentration and the SOFA score improved the diagnostic accuracy in distinguishing patients with and without septic encephalopathy, and an elevated serum Tau protein was a predictor of 28-day mortality in severe sepsis (Zhao et al., 2019). Although most data showing sepsis to induce the accumulation of amyloid-β and p-Tau in the brain are from animal models, several pieces of evidence support the hypothesis that sepsis promotes amyloid-β and p-Tau accumulation in the brain of humans (Sekino et al., 2022).

#### 2.2.5. Neurofilament light chains (NfL) and neurofilament heavy chains (NfH)

Neurofilaments are expressed exclusively in neurons. Here they are concentrated in large axons. They are released upon axonal degeneration. Plasma NfL concentrations were higher in patients with septic encephalopathy than in patients with sepsis without encephalopathy and in patients without sepsis. Repeated measures of NfL during sepsis appeared particularly useful, because they could be an indicator of progressive cerebral injury. NfL levels significantly increased in patients with brain dysfunction over time and remained low in patients without brain dysfunction. NfL levels correlated with the severity of acute septic encephalopathy and with a poorer functional outcome after 100 days (Ehler et al., 2019a). In 150 patients with community-acquired pneumonia, confused patients had higher NfL concentrations in serum compared to non-confused patients with comparable disease severity. In a logistic regression analysis, NfL levels in serum were a strong predictor for an unfavorable outcome (Chung et al., 2023).

High concentrations of NfL were measured in the CSF of septic encephalopathy patients. CSF NfL levels were higher in fatal cases than in survivors, and correlated with days until death (Ehler et al., 2019a).

Although NfL values show inter-individual variability in healthy persons, values increase with age, impaired renal and liver function, and are elevated in neurodegenerative diseases and in critical-illness peripheral neuropathy (Bircak-Kuchtova et al., 2023). Nevertheless, measurement of NfL in plasma or serum is becoming increasingly popular as an indicator of long-term sequelae in septic encephalopathy (Bircak-Kuchtova et al., 2023).

### 2.3. Routine cerebrospinal fluid analysis

Routine CSF parameters in septic encephalopathy usually are either normal or only slightly abnormal (Bitsch et al., 1996; Tauber et al., 2021). In a recent study, each of 12 patients with septic encephalopathy had a normal leukocyte count (normal ≤4/μl), and only 2 of 12 patients had a mild elevation of the CSF total protein concentration (Ehler et al., 2019a,b). In another series of patients with septic encephalopathy, CSF protein often was slightly elevated, without elevation of CSF leukocytes, and no signs of intrathecal immunoglobulin synthesis were noted (Chung et al., 2020).

Conversely, in 11 of 12 patients with stroke-like symptoms caused by septic-embolic encephalitis, in CSF analysis some kind of inflammation was detectable. Abnormalities observed included CSF leukocytosis from 2 to 496 cells/μl (median 247 cells/μl), elevated protein content from 310 to 3,224 mg/L (median 923 mg/L), and elevated lactate concentrations from 2.2 to 6.7 mmol/L (median 3.5 mmoL/L) (Bitsch et al., 1996). In patients with septic-metastatic encephalitis (*n* = 6), inflammation in the CSF on average was more pronounced than with septic-embolic encephalitis, and CSF pleocytosis was observed in all patients. The CSF leukocyte counts ranged from 17 to 10,000 cells/μl (median 473/μl), the CSF protein content from 478 to 4,249 mg/L (median 739 mg/L), and the CSF lactate from 2.8 to 7.9 mmoL/L (median 3.0 mmoL/L) (Bitsch et al., 1996).

Typically, a lumbar puncture is performed in patients with septic encephalopathy with delirium or mental obtundation to exclude meningitis or meningoencephalitis (Tauber et al., 2021). Nuchal rigidity is not a sensitive and not a specific sign of meningeal inflammation. Particularly in elderly and very young patients with bacterial meningitis, fever and nuchal rigidity can be absent. Therefore, in the elderly, definitive exclusion of meningitis or meningoencephalitis relies on CSF analysis (Miller and Choi, 1997; Tauber et al., 2021).

Since in humans proteins of the size of IgG need approximately 4 days to equilibrate between blood and CSF (Frick and Scheid-Seydel, 1958), CSF analysis in critically ill patients can be complicated by rapid volume shifts, by plasmapheresis and immune absorption, and by the intravenous administration of albumin or immunoglobulins (Djukic et al., 2022). The rapid correction of a strong intravascular volume deficit by intravenous electrolyte or glucose solutions can mimic an impairment of the blood-CSF barrier. Similarly, removal of a large fraction of the immunoglobulins in plasma by immune absorption can mimic abnormal intrathecal antibody synthesis in the Reiber-Felgenhauer diagrams. Conversely, the intravenous infusion of IgG can lead to an apparent disappearance of an intrathecal IgG synthesis in the Reiber-Felgenhauer diagrams, and the intravenous infusion of high doses of albumin can lead to an apparent disappearance of a blood-CSF barrier dysfunction and an imaginary intrathecal immunoglobulin synthesis (Djukic et al., 2022). Moreover, the entry of blood into the CSF can affect the use of Reiber-Felgenhauer nomograms for the detection of intrathecal immunoglobulin synthesis. Here, the IgM nomogram is most sensitive, and even a blood contamination by 0.1% can cause an apparent intrathecal IgM synthesis (Djukic et al., 2022).

A single lumbar puncture often is necessary to rule out meningitis or encephalitis. Repeated lumbar punctures, although desirable in order to analyze the course of the concentrations of biomarkers of septic encephalopathy in CSF, often are not feasible because of the poor health conditions of patients with septic encephalopathy including abnormalities of coagulation. The biomarkers successfully used in plasma/serum have been only occasionally employed in CSF (e.g. Ehler et al., 2019a,b), although their CSF concentrations probably reflect neural injury more accurately than the respective plasma/serum concentrations.

A suggestion for parameters which can be measured in plasma/serum or CSF in routine clinical laboratories and may either serve as indicators of the severity of sepsis or surrogate markers for neuronal, axonal and astrocyte injury is listed in Table 1.

**Table 1:** Suggested biomarkers in plasma to monitor the course of septic encephalopathy.*

Parameter	Significance	Serum/plasma	CSF	Specific for encephalopathy/encephalitis	Reference
*General markers of the severity and outcome of sepsis*					
C-reactive protein (CRP)	Quantification of inflammation by a widely available and inexpensive method	++	Ø	No	McGrane et al. (2011), Siebert et al. (2017), and Zacharakis et al. (2023)
Procalcitonin (PCT)	Quantification of inflammation, discriminates more accurately between bacterial/fungal infection and viral/autoimmune inflammation, higher PCT levels in Gram-negative than in Gram-positive sepsis, reacts more quickly than CRP	++	Ø	No	Leli et al. (2015) and Falcone et al. (2023)
NT-proCNP	Increased in septic encephalopathy, specific for endothelial cells	+	?	No	Koch et al. (2011) and Ehler et al. (2019a,b)
IL-6, IL-8, IL-10 and TNF-α	Established for the assessment of outcome in sepsis	++	(+)	No	Ishikawa et al. (2021) and Xiao et al. (2022)
*Indicators of nervous tissue injury*					
Neuron-specific enolase (NSE)	Classical marker of neuronal injury, plasma concentrations strongly increased by hemolysis	++	+	Partially, also present in erythrocytes and thrombocytes	Yao et al. (2014)
Tau protein (Tau)	Classical marker of neuronal injury	++	+	Yes	Zhao et al. (2019)
S100B protein (S100B)	Classical marker of glial injury	++	+	Yes	Yao et al. (2014)
Glial fibrillary acidic protein (GFAP)	Indicator of poor outcome in septic encephalopathy	++	?	Yes	Yan et al. (2019) and Wu et al. (2019)
Neurofilament light chain (NfL)	New marker of axonal injury	++	+	Yes	Ehler et al. (2017, 2019a,b) and Bircak-Kuchtova et al. (2023)

*These markers are suggested by the authors on the basis of the data reviewed (McGrane et al., 2011; van den Boogaard et al., 2011; Tomasi et al., 2017; El Shimy et al., 2018; Ehler et al., 2019a,b; Visitchanakun et al., 2020; Wu et al., 2019; Zhao et al., 2019; Tauber et al., 2021; Bircak-Kuchtova et al., 2023) and the availability of the assays in clinical practice. Measurement of NSE, amyloid-β, Tau, S100B and NfL in CSF may be more sensitive or specific than determination in plasma, yet in septic encephalopathy patients CSF often is not available, and repeated CSF analyses are rarely possible.

### 2.4. Functional methods to diagnose and monitor septic encephalopathy

#### 2.4.1. Electroencephalography (EEG) and sensory evoked potentials (SEP)

EEG is a very sensitive non-invasive tool to detect septic encephalopathy. The severity of EEG abnormalities can be classified as predominant slow alpha activity, predominant theta activity, predominant delta activity, and burst-suppression activity. The incidence of EEG abnormalities during sepsis ranged from 12 to 100% for background abnormality and 6 to 12% for the presence of triphasic waves. The severity of EEG abnormalities correlated with outcome (Hosokawa et al., 2014; Azabou et al., 2015). EEG can exclude or confirm non-convulsive status epilepticus which requires treatment with antiepileptics (Pantzaris et al., 2021). The specificity of EEG for the diagnosis of septic encephalopathy is low, and sedatives can mimic abnormalities encountered in severe septic encephalopathy (Dumbuya et al., 2023).

SEPs measure the brain’s response upon sensory stimulation. They are less susceptible to sedation, but the recording of SEPs is cumbersome in intensive care units. Slowing of SEP latencies has been described in septic encephalopathy, but data correlating SEPs with outcome are sparse (Tauber et al., 2021; Dumbuya et al., 2023).

#### 2.4.2. Transcranial Doppler ultrasonography

Continuous or intermittent transcranial Doppler ultrasonography has been used to monitor septic encephalopathy. The pulsatility index [PI = (systolic velocity - diastolic velocity)/mean velocity] and the cerebral blood flow index (CBFi = mean arterial pressure × 10/1.47^PI^) were calculated from measurements in the middle cerebral artery. The authors concluded that “cerebral perfusion disturbance observed with transcranial Doppler could explain clinical symptoms of sepsis-associated encephalopathy” (Pierrakos et al., 2014). An impairment of cerebrovascular autoregulation by sepsis was associated with sepsis-associated delirium suggesting that an abnormal cerebral autoregulation contributes to the development of septic encephalopathy (Schramm et al., 2012). In 45 children with septic encephalopathy, the pulsatility index and resistive index was higher than in children without septic encephalopathy. An increased cerebrovascular resistance was associated with mortality (Algebaly et al., 2020). Since head movements can compromise the transcranial ultrasound signal, transcranial Doppler sonography appears particularly useful in sedated or spontaneously comatose patients with septic encephalopathy (Robba et al., 2018).

#### 2.4.3. Near-infrared spectroscopy

In a small selected group of non-sedated patients with septic encephalopathy, continuous monitoring based on near-infrared spectroscopy and measurement of the mean arterial blood pressure improved cerebral perfusion (Rosenblatt et al., 2020). In 48 patients with septic encephalopathy, the minimum regional cerebral oxygen saturation in the forebrain was lower in non-survivors than in survivors, and the variation of the regional oxygen saturation was greater in patients with delirium (Peng et al., 2021).

#### 2.4.4. Imaging techniques for the detection and monitoring of septic encephalopathy

##### 2.4.4.1. Magnetic resonance imaging

Larger cerebral ischemic or hemorrhagic lesions or a combination of both are indicative of septic-embolic encephalitis, and cerebral microabscesses constitute septic-metastatic encephalitis (Figure 3). In septic encephalopathy, routine MRI including diffusion-weighted imaging often is normal. However, in a series of 13 patients with septic encephalopathy, MRI detected diffuse axonal injury in 9 and small ischemic lesions in 3 patients (Ehler et al., 2017). The use of diffusion tensor imaging (DTI) probably will increase the sensitivity of MRI in comparison with conventional MRI techniques to detect diffuse axonal injury (Ehler et al., 2017).

In 156 patients with septic encephalopathy, 14 (8.9%) on cMRI had findings compatible with the posterior reversible encephalopathy syndrome (PRES) (Figure 4). Patients with PRES often showed lesions in atypical regions, including frontal lobes, the corpus callosum, and the basal ganglia (Orhun et al., 2022). PRES appears to be a typical feature of sepsis caused by Gram-positive organisms (Bartynski et al., 2006). Because of the overall good outcome of PRES, autopsy findings are rare and include swollen vascular endothelium, vasogenic edema and diffuse axonal injury (Willard et al., 2018).

##### 2.4.4.2. Positron emission tomography (PET) and single photon emission tomography (SPECT)

**Figure 5:**
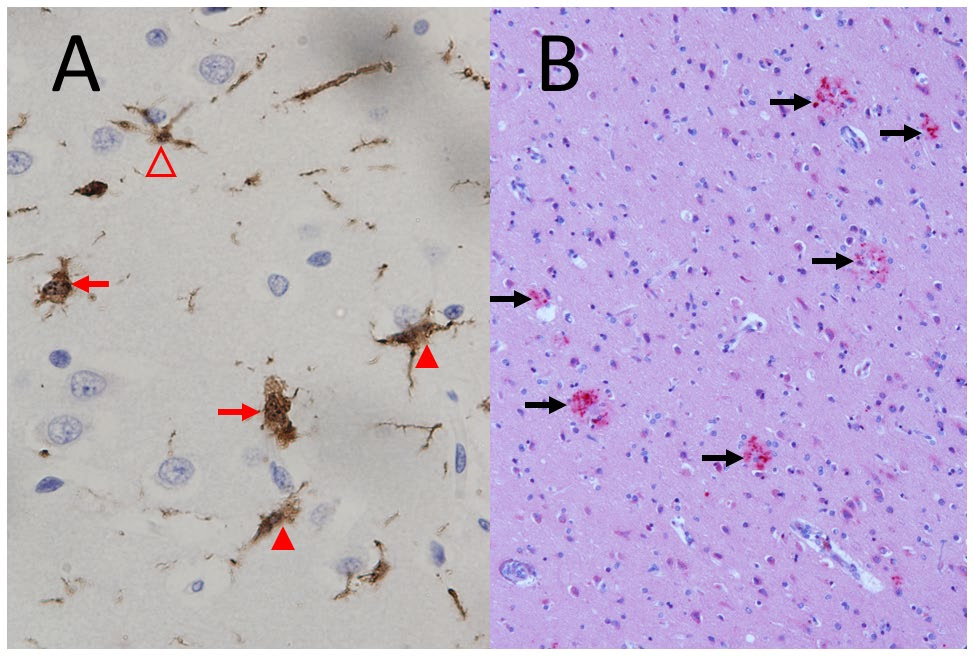
Microglial activation and diffuse axonal injury typical for septic encephalopathy. **(A)** Strong microglial activation in a fatal case of *Neisseria meningitidis* sepsis (young woman), immunohistochemistry by polyclonal rabbit anti-Iba-1 (ionized calcium-binding adaptor molecule 1) antibodies (1:400, Wako, Neuss, Germany) counterstained with hemalum. Please note the strongly activated ameboid microglia without processes (red arrows) and the moderately activated microglia with shortened processes of increased thickness (closed arrowheads) and the mildly activated microglial cell with preserved processes (open arrowhead) (objective: x40). **(B)** Diffuse axonal injury (red, marked by black arrows) in a 75 years old man with liver cirrhosis, sepsis and endocarditis, immunohistochemistry by a mono.clonal mouse antibody against amyloid-β precursor protein (APP) (1:2000, Chemicon, Temecula, CA, USA) counterstained with hemalum (objective: x20).

PET and SPECT are ideal techniques to study microglial activation (Figure 5), one of the hallmarks of septic encephalopathy. To our knowledge, however, microglial activation during septic encephalopathy has not been investigated in humans by these methods. One reason may be the comparatively long study time, which impedes the use of these technique in critically ill patients.

In an experimental rat sepsis model, PET demonstrated a reduced cerebral glucose uptake (Semmler et al., 2008). After LPS injection in mice, an increased [18F]fluorodeoxyglucose (FDG) uptake was noted, whereas [99mTc]hexamethylpropylene amine oxime (HMPAO) SPECT suggested brain hypoperfusion. Increased microglial activity was visualized by [125I]6-chloro-2-(4′-iodophenyl)-3-(N,N-methylethyl)imidazo[1,2-a]pyridine-3-acetamide (CLINME) SPECT (Szöllősi et al., 2018).

## 3. Limitations of this review

The unambiguous diagnosis of septic encephalopathy can be complicated, because electrolyte and metabolite disturbances (in particular hyponatremia, hypernatremia and hypercalcemia, hypo- and hyperglycemia, lactate acidosis) and renal or hepatic failure, which may or may not be related to sepsis in an individual patient, can cause similar clinical symptoms. For this reason, there is a strong need to clearly differentiate between neural injury caused by septic encephalopathy and damage caused by other mechanisms.

Considering the high variability in human samples, investigations with low patient numbers must be interpreted cautiously. Although the incidence of septic encephalopathy is highest in old age, there is a lack of studies specifically addressing the utility of the available biomarkers in older persons.

## 4. Conclusion and suggestions for future research

**Figure 6:**
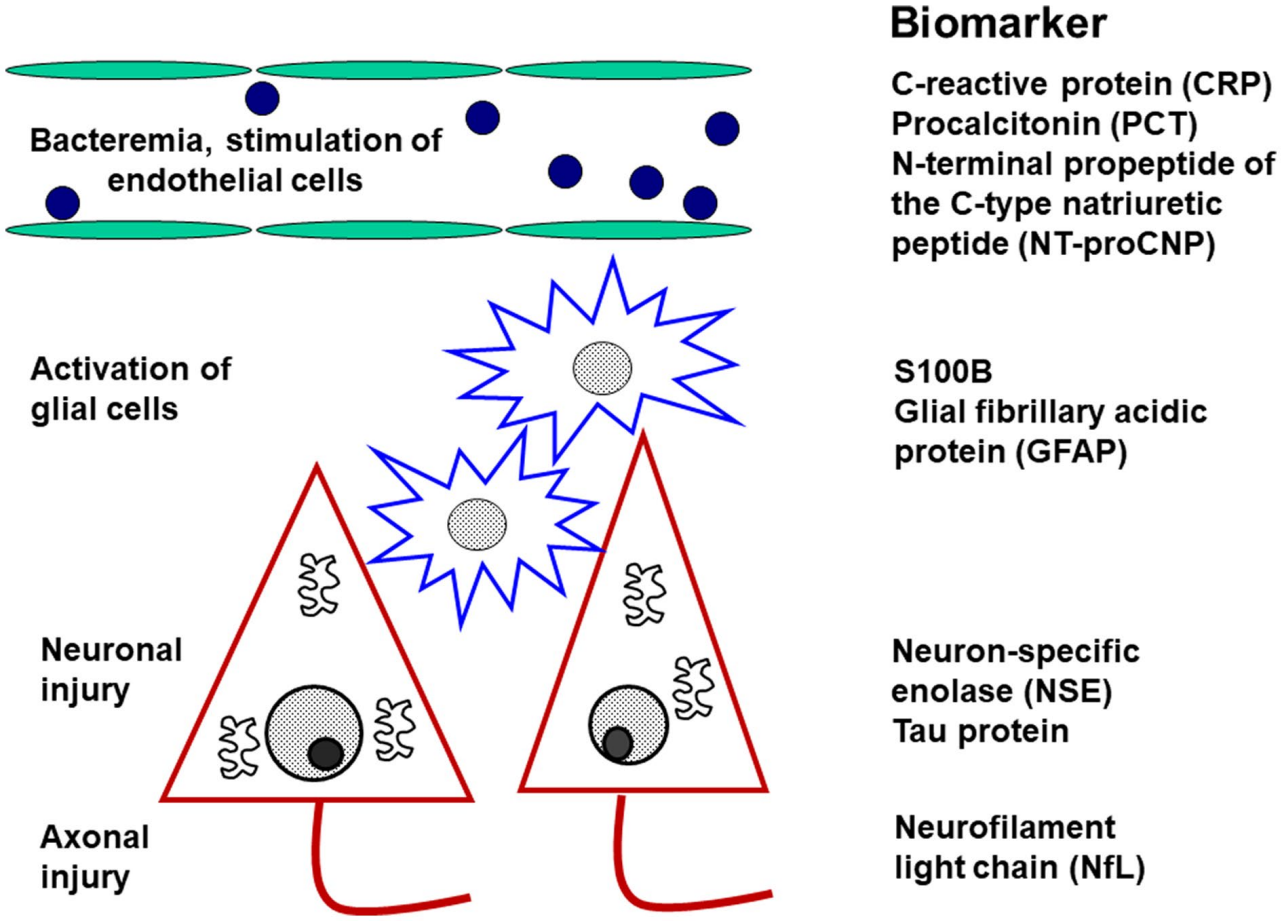
Diagnostic biomarkers of activation and injury in septic encephalopathy. The biomarkers are arranged according to the site of origin. The markers of endothelial activation are not specific for the brain, whereas the markers of glial activation (and injury) and of neuronal or axonal injury are relatively specific for the central nervous system.

Basically, the pathology of septic encephalopathy can be characterized by three steps, and biomarkers of each step should be defined (Ehler et al., 2020; Figure 6):

1. Systemic inflammation causes dysfunction of the cerebral endothelium. Since C-type natriuretic peptide is predominantly expressed in by endothelial cells and released during sepsis (Koch et al., 2011) and septic encephalopathy from endothelial cells, its N-terminal propeptide (NT-proCNP) may serve as an indicator of injury to the cerebrovascular endothelium (Ehler et al., 2020). Whether it is really more specific for cerebrovascular dysfunction than procalcitonin or C-reactive protein remains to be determined.

2. The key event causing neuronal injury in septic encephalopathy is the activation of microglial cells (Yan et al., 2022). In the absence of plasma/serum biomarkers of microglial activation, S100B as a marker of glial cell damage has been proposed for the monitoring of microglial activity (Ehler et al., 2020). A specific marker truly quantifying microglial activity would represent a major advance for the monitoring of sepic encephalopathy.

3. Death of neurons and damage to axons and synapses is the morphological correlate of long-term neurocognitive deficits after septic encephalopathy. Although primarily an indicator of axonal injury, neurofilament light chains (NfL) have been proposed as an indicator of neuronal damage (Ehler et al., 2020). We suggest that it may be advantageous to also determine neuron-specific enolase (NSE) in plasma/serum. When hemolytic samples are excluded, NSE already established as a prognostic marker in hypoxic brain injury (Sharma et al., 2022; Kim et al., 2023) probably is a valuable tool for the quantification of neuronal injury in septic encephalopathy of older persons.

Among new biomarkers for septic encephalopathy not yet established in clinical practice, brain-specific microRNAs appear most promising (Suster and Feng, 2021; Antonakos et al., 2022; Li et al., 2023; Yuechen et al., 2023).

## Author contributions

RN conceived the study. SS, DD, ST, and RN searched the literature and analyzed previously published data, and designed the figures. SS wrote the first draft of the manuscript. All authors contributed to the Discussion, reviewed and approved the final version of this manuscript.

## Funding

This work was funded by the Open Access Publication Fund of the Georg-August University of Göttingen and the German Society of Geriatrics.

## Conflict of interest

The authors declare that the research was conducted in the absence of any commercial or financial relationships that could be construed as a potential conflict of interest.

## Publisher’s note

All claims expressed in this article are solely those of the authors and do not necessarily represent those of their affiliated organizations, or those of the publisher, the editors and the reviewers. Any product that may be evaluated in this article, or claim that may be made by its manufacturer, is not guaranteed or endorsed by the publisher.
